# Central Muscarinic Cholinergic Activation Alters Interaction between Splenic Dendritic Cell and CD4^+^CD25^-^ T Cells in Experimental Colitis

**DOI:** 10.1371/journal.pone.0109272

**Published:** 2014-10-08

**Authors:** Peris Munyaka, Mohammad F. Rabbi, Valentin A. Pavlov, Kevin J. Tracey, Ehsan Khafipour, Jean-Eric Ghia

**Affiliations:** 1 University of Manitoba, Department of Immunology and Internal Medicine section of Gastroenterology, Winnipeg, Manitoba, Canada; 2 Center for Biomedical Science, The Feinstein Institute for Medical Research, Manhasset, New York, United States of America; 3 University of Manitoba, Department of Animal Sciences, Winnipeg, Manitoba, Canada; 4 University of Manitoba, Inflammatory Bowel Disease Clinical and Research Centre, Winnipeg, Manitoba, Canada; Duke University Medical Center, United States of America

## Abstract

**Background:**

The cholinergic anti-inflammatory pathway (CAP) is based on vagus nerve (VN) activity that regulates macrophage and dendritic cell responses in the spleen through alpha-7 nicotinic acetylcholine receptor (a7nAChR) signaling. Inflammatory bowel disease (IBD) patients present dysautonomia with decreased vagus nerve activity, dendritic cell and T cell over-activation. The aim of this study was to investigate whether central activation of the CAP alters the function of dendritic cells (DCs) and sequential CD4^+^/CD25^−^T cell activation in the context of experimental colitis.

**Methods:**

The dinitrobenzene sulfonic acid model of experimental colitis in C57BL/6 mice was used. Central, intracerebroventricular infusion of the M1 muscarinic acetylcholine receptor agonist McN-A-343 was used to activate CAP and vagus nerve and/or splenic nerve transection were performed. In addition, the role of α7nAChR signaling and the NF-kB pathway was studied. Serum amyloid protein (SAP)-A, colonic tissue cytokines, IL-12p70 and IL-23 in isolated splenic DCs, and cytokines levels in DC-CD4^+^CD25^−^T cell co-culture were determined.

**Results:**

McN-A-343 treatment reduced colonic inflammation associated with decreased pro-inflammatory Th1/Th17 colonic and splenic cytokine secretion. Splenic DCs cytokine release was modulated through α7nAChR and the NF-kB signaling pathways. Cholinergic activation resulted in decreased CD4^+^CD25^−^T cell priming. The anti-inflammatory efficacy of central cholinergic activation was abolished in mice with vagotomy or splenic neurectomy.

**Conclusions:**

Suppression of splenic immune cell activation and altered interaction between DCs and T cells are important aspects of the beneficial effect of brain activation of the CAP in experimental colitis. These findings may lead to improved therapeutic strategies in the treatment of IBD.

## Introduction

Inflammatory bowel diseases (IBD) are idiopathic chronic, recurrent intestinal disorders of complex pathogenesis, which include Crohn's disease (CD) and (UC) [1]. The IBD etiopathogenesis is multifactorial, involving an aberrant immune response to gut bacterial antigens that develop in genetically predisposed individuals [1]. Dysregulated immune responses in the context of IBD have been therapeutically targeted by biologicals (*i.e.* anti- tumor necrosis factor (TNF)-α), corticosteroids and thiopurines [2]. IBD is reflected by a Th1 and Th17 immune response [3], [4], where the Th2 response seems to play only a minor role [5]. In line with this paradigm new therapeutics have been currently studied in clinical settings of IBD [6].

The cholinergic anti-inflammatory pathway (CAP) controls the production of different Th1 and Th17 cytokine in several inflammatory models, including experimental colitis [7], [8]. Increased vagus nerve activity through the release of acetylcholine (ACh) in the reticuloendothelial system has been associated with decreased immune cell activation and altered cytokine release [9], [10]. This effect is mainly mediated through the alpha-7 nicotinic acetylcholine receptor (α7nAChR) signaling in antigen presenting cells [11]. Cellular and molecular mechanisms underlying the CAP and the role of the spleen have been actively investigated. In addition to electrical vagus nerve (VN) stimulation [12], this physiological mechanism can be activated in the CNS by using galantamine, a centrally-acting acetylcholinesterase inhibitor [9], [13], [14]. We have previously reported that the central activation of the CAP inhibits acute inflammation in a murine models of colitis resembling UC and CD [9]. In this context, treatments with galantamine ameliorated acute colitis through a dendritic cell (DC)-mediated mechanism and major histocompatibility complex (MHC) II regulation. Moreover, in line with the role of α7nAChR in mediating anti-inflammatory cholinergic signals, we have demonstrated that an α7nAChR agonist regulates DC interleukin (IL)-12p40 release [9]. This is consistent with data demonstrating that *in vitro* mature spleen DCs that are exposed to nicotine produce decreased levels of IL-12p40 [15].

IBD is characterized by mucosal recruitment of a variety of immune inflammatory cells including DCs [1]. A hallmark of IBD is a marked accumulation of myeloid cells particularly monocytes and DCs. Accordingly, selective depletion or adhesion blockade of DCs by Diphtheria Toxin or anti-IL-12 treatment suppress DSS-colitis [16], [17]. DCs are found in the spleen and the intestine where they are present at the level of the lamina propria, Peyer's patches and lymphoid follicles and outside the intestine within the mesenteric lymph nodes (MLN) [18]. These DCs are in an ‘immature’ state and are unable to stimulate T cells. Although these DCs lack the required accessory signals for T cell activation, such as cluster of differentiation (CD) 40, they are in position to capture antigens (Ag). Once primed, they migrate to the secondary lymphoid compartments (MLN or spleen) to present Ag-peptide complexes to naïve CD4^+^ T cells and CD8^+^ cytotoxic T cells to initiate pathology or tolerance *via* an increase in expression of MHC II, CD40, CD80 and CD86 co-stimulatory molecules and an increase in IL-12p70 and IL-23 expression. No only the expression of the co-stimulating factors can alter T-cell priming, but the cytokine profile released by the DCs can potentially regulate T-cell priming.

Although, recent findings have highlighted a key role of the splenic DCs in mediating vagus nerve anti-inflammatory signaling during experimental colitis [9], the exact role of a vagus nerve -to spleen DCs anti-inflammatory axis in the regulation of intestinal inflammation remained to be further characterized. Thus, the present study examined the role of the vagus in two models of colitis induced: i) a lymphocyte-dependent model by intracolonic administration of 2, 4-dinitrobenzene sulfonic acid (DNBS) [19] and ii) a semi lymphocyte-independent self-limiting model of erosive colonic injury and inflammation through oral administration of dextran sodium sulfate (DSS) [20], [21]. Here we studied whether, in the context of experimental colitis, central activation of the CAP by an M1mAChR agonist alters T cell priming *via* splenic DCs, through an effect mediated by the vagus and the splenic nerve and the release of DC cytokine. We demonstrate that central administration of McN-A-343 significantly ameliorates disease severity and inhibits inflammation in the context of experimental colitis *via* a decrease of splenic T cell priming. This therapeutic efficacy depends on the integrity of the vagus and the splenic nerve and is mediated through modulation of the functional interaction between DCs and CD4^+^CD25^−^T cell *via* an α7nAChR and NF-κB signaling, demonstrating the potential effect of central activation of the CAP in the context of antigen presenting cell and the important role of cytokine release by DC in the regulation of T-cell priming.

## Materials and Methods

### Animals

Male C57BL/6 mice (7–9 weeks old) were purchased from Charles Rivers (Canada) and maintained in the animal care facility at the University of Manitoba under specific pathogen-free conditions. No differences in food intake or body weight were observed between the groups after vagotomy procedure.

### Surgical procedures & drug treatments

**Figure 1:**
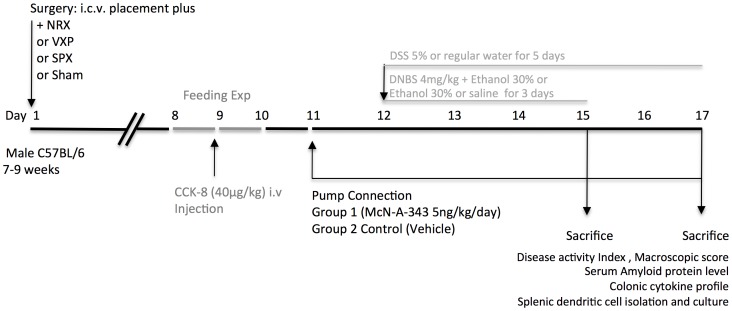
Experimental protocol. On day 1, mice were anaesthetized using ketamine (150 mg/kg, i.p) and xylazine (10 mg/kg, i.p) and i.c.v. Placement of the cannula, splenic neurectomy (NRX), subdiaphragmatic bilateral vagotomy (VXP), or splenectomy (SPX) were performed. Mice were allowed to recover for 10 days. Completeness of vagotomy was verified using the CCK-8 satiety test starting on day 8. On day 11, pharmacological treatment started: micro-osmotic pumps were connected to cannula and filled with vehicle or the McN-A-343. On day 12, colitis was induced using two specific models of gut inflammation: 2,4-dinitrobenzene sulfonic acid (DNBS) or dextran sulfate sodium (DSS) models. Colitis was induced by intra-rectal administration of 100 µl of 4 mg of DNBS solution in 30% ethanol and the mice left for 3 days. Alternatively, DSS was added to the drinking water in a final concentration of 5% (wt/vol) for 5 days. Controls were all time-matched and consisted of mice that received normal drinking water only. Colonic samples were collected 3 and 5 days post-DNBS and DSS activation respectively.

Mice were anaesthetized using ketamine (150 mg/kg, i.p; Wyeth, St Laurent, Canada) and xylazine (10 mg/kg, i.p; Bayer Inc. Toronto, Canada). I.c.v. implantation of the cannula, splenic neurectomy (NRX), subdiaphragmatic bilateral vagotomy (VXP), or splenectomy (SPX) were performed on the same day [9] (Figure 1). The completeness of vagotomy was verified using the CCK-8 satiety test [22], [23] and *via* post-mortem inspection of vagal nerve endings using microscopic observation by using a Bielschowsky silver staining [24]. The completeness of neurectomy was verified post-mortem by noradrenaline enzyme-linked immunosorbent assay in sham-operated and NRX animals; a success rate of 94% was achieved (data not shown). Mice were allowed to recover for 10 days. One day before initiation of colitis pharmacological treatment started: micro-osmotic pumps (Alzet, Cupertino, CA) were connected to cannula and filled with vehicle (saline) or the McN-A-343 (5 ng/day, Sigma, Mississauga, Canada) and placed as previously described [9].

### Induction of colitis

Two specific models of gut inflammation were used. First we used the DNBS model of colitis. For the DNBS study, mice were anaesthetized with Isoflurane® (Abbott, Toronto, Canada). A 10 cm long PE-90 tubing (ClayAdam, Parisppany, NJ), attached to a tuberculin syringe (BD, Mississauga, Canada), was inserted (intrarectally) 3.5 cm into the colon. Colitis was induced by intra-rectal administration of 100 µl of 4 mg of DNBS solution (ICN Biomedical Inc., Aurora, OH) in 30% ethanol (Sigma) and the mice left for 3 days [20]. Mice were killed on day 3 and samples were taken. Control mice (without colitis) received saline administration. As no effect of the treatment and surgery was visible using the 30% ethanol group, only the DNBS group is presented. Alternatively, we used a second model of epithelial erosion using dextran sulfate sodium (DSS) (molecular weight [MW], 40 kilodaltons; MP Biomedicals, Soho, OH). DSS was added to the drinking water in a final concentration of 5% (wt/vol) for 5 days [21]. Controls were all time-matched and consisted of mice that received normal drinking water only. Mean DSS consumption was noted per cage each day. Mice with colitis were supplied with 6% sucrose (Sigma) in drinking water to prevent dehydration.

### Characterization of inflammation

Colonic samples were collected 3 and 5 days post-DNBS and DSS activation respectively, and blood was collected by intracardiac puncture under isoflurane (Abbot) anaesthesia. Serum amyloid protein-A (SAP) was used as a marker for systemic inflammation and was determined using ELISA commercial kit (R&D Systems, Minneapolis, MN). Colonic samples were homogenized in 700 microliter of Tris-HCl buffer containing protease inhibitors (Sigma). Samples were centrifuged for 30 minutes, and the supernatant was frozen at 80°C until assay. Cytokine levels (TNF-α, IL-6, IL-1 β, IFN-γ, IL-17, IL-12p70, IL-23 and IL-4) were determined using an enzyme-linked immunoabsorbant assay commercial kit (R&D Systems).

### Isolation of splenic CD11c^+^ cells and culture. Isolation of splenic CD11c^+^ cells and culture

3 or 5 days post-activation associated with DNBS or DSS colitis respectively, the spleens were digested in 2 mg/ml^−1^ collagenase D (Roche Diagnostics, Meylan, France) in RPMI 1640 (Life Technologies, Grand Island, NY) for 30 min at 37°C. EDTA (Sigma) at 5 mM was added during the last 5 min to disrupt DC-T cell complexes, and the cell suspension was filtered. Total splenocytes after RBC lysis with ACK lysis buffer (150 mM NH4Cl, 10 mM KHCO3, 0.1 mM EDTA; Life Technologies) were incubated with CD11c^+^ microbeads (Miltenyi Biotec, Auburn, CA) for 15 min at 48°C. The cells were then washed, resuspended in cell separation buffer (Dulbecco's Phosphate-Buffered Saline [D-PBS] without Ca21 and Mg21 containing 2% FBS and 2 mM EDTA, (Life Technologies) and passed through magnetic columns (Miltenyi Biotec) for positive selection. After passing consecutively through two columns, the collected splenic CD11c^+^ cell preparations showed greater than 95% purity. Splenic CD11c^+^ cell isolated from different groups of mice were cultured in complete RPMI 1640 medium (Life Technologies) containing 10% heat-inactivated FBS, 25 mg/ml^−1^ gentamicin, 2 mM L-glutamine in 12-well plates at 1.10^+6^ cells/well for 24 hrs, and the supernatants were measured for IL-12p40, IL-6 and TNF-α by ELISA (R&D Systems). To characterize the CD11c^+^ cellular and intracellular pathway, in a separate set of experiment the α7nAChR agonist GTS-21 (100 µM; Sigma) or methyllycaconitine (a specific a-7 nicotinic antagonist, 10 µM; Sigma) or betulinic acid (a specific NF-κB activator, 10 µM; Sigma) or BAY 11-7082 (a specific NF-κB inhibitor, 10 µM; Sigma) were added to medium.

### CD4^+^CD25^-^ T Cell co-culturing with CD11c^+^ DCs

Spleen were removed from mice and gently meshed in DMEM containing 10% FBS (Life Technologies) to prepare for single cell suspensions. CD4^+^/CD25^−^ T cells were isolated by the CD4^+^CD25^−^ T cell isolation kit (Miltenyi Biotec) according to manufacturer's instruction. The purity of CD4^+^CD25^−^ populations was around 90%. CD11c^+^ DCs isolated from colitic mice receiving the different treatments were cultured for 24 hrs in the presence or absence of GTS-21 (a specific α7nAChR agonist, 100 µM) before medium was washed and co-cultured with CD4^+^CD25^−^ T cell isolated from naïve mice at ratio of 1∶3 (DCs:T cells) [25] in plates coated with 10 µg/ml^−1^ of anti-CD3 and 2 µg/ml^−1^ of anti-CD28. In neutralization experiments, these cultures were treated with 10 µg/ml anti-IL-12p35 or anti-IL-23p19 (R&D Systems) neutralizing mAb to block the potential activities of endogenous sources of these cytokines. In a separated set, recombinant (r) IL-12p70 or rIL-23 protein (25 ng/ml^−1^; R&D Systems) were added to the cell culture medium. Cell culture supernatants were collected at 24 hrs, and interferon-gamma (IFN-γ), IL-4 and IL-17 levels were analyzed by ELISA (R&D Systems).

### Statistical analysis

Results are presented as means ± SEMs. Statistical analysis was performed using one or two way ANOVA followed by the Tukey-Kramer multiple comparisons *post hoc* analysis and a *P* value of <0.05 was considered significant with n = 8 to 12 depending on the groups tested (Prism 4, GraphPad, La Jolla, CA).

### Ethical considerations

All experiments were approved by the University of Manitoba animal ethics committee (10-073) and conducted under the Canadian guidelines for animal research.

## Results

### Central muscarinic cholinergic activation decreases macroscopic score and colonic length via the vagus nerve and the splenic nerve

**Figure 2:**
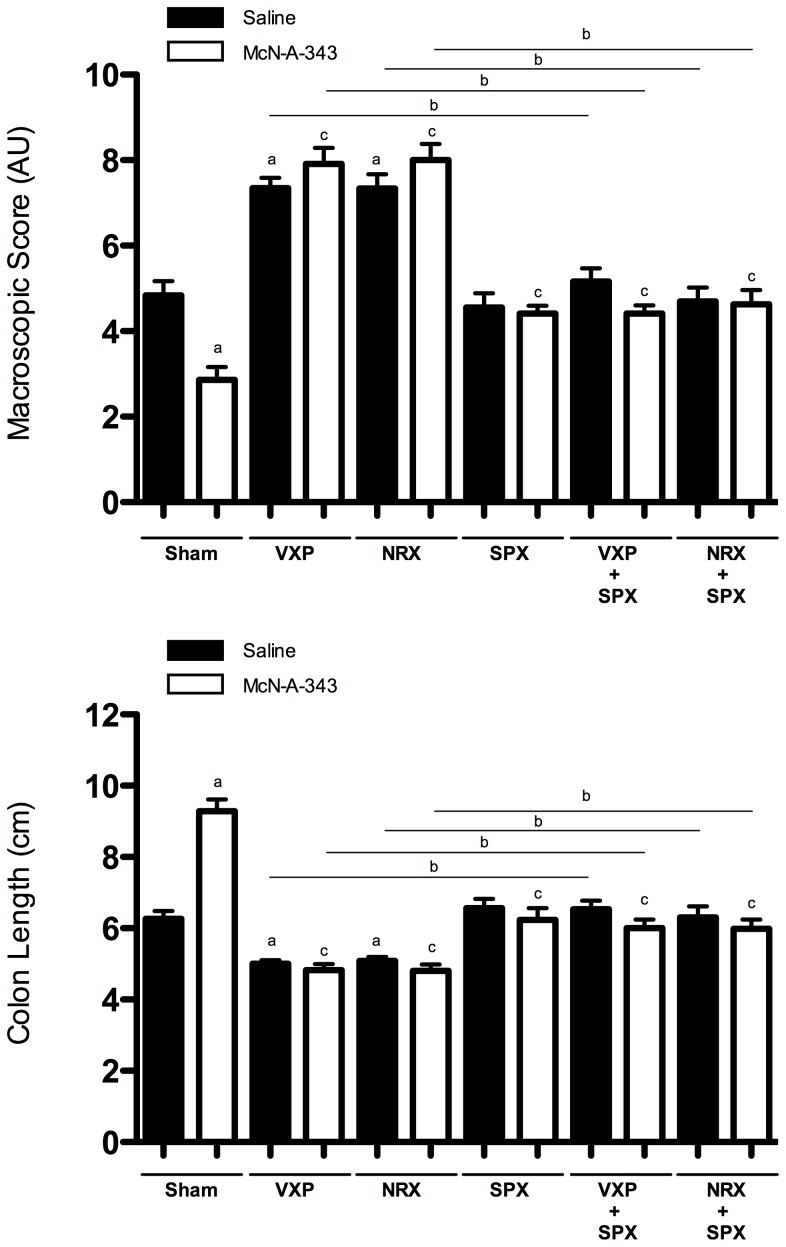
Central administration of a M1mAchR agonist alleviates macroscopic markers of 2, 4-dinitrobenzene sulfonic acid (DNBS)–induced colitis through vagus nerve and splenic nerve signaling to the spleen. Vagotomy (VXP) and/or splenectomy (SPX), splenic neurectomy (NRX) and/or splenectomy (SPX) were performed 10 days prior to initiating McN-A-343 (5 ng/kg/day, i.c.v.) treatment and/or colitis induction as described in Material and Methods. *Sham represents data obtained in sham SPX mice, because no significant differences were determined between this group and any other sham group of animals; ***A***: Macroscopic score and ***B***: colon length were evaluated. Values are shown as means ± SEM. Samples were collected on day 3 post-DNBS induction; mice per group ≥8. ^a^ *P*<0.05 as compared to sham-saline-DNBS-treated group, ^b^ *P*<0.05 as compared to VXP-DNBS-treated group or NRX-DNBS-treated group respectively, ^c^ *P*<0.05 as compared to sham-McN-A-343-DNBS-treated group.

First we examined the relation between the central stimulation and the development of experimental colitis. Infusion with McN-A-343 (5 ng/kg/day), started one day before induction of DNBS colitis, decreased the macroscopic scores and increased the length of colon at day 3 (Figure 2). In addition to its efficacy in DNBS colitis, McN-A-343 treatment decreased the severity of DSS colitis (Table 1). These results confirmed the disease-alleviating and counter-inflammatory effects of centrally-acting M1mAChR agonist treatments in the two models of colitis. To further study whether the beneficial effects of central cholinergic activation in the context of colitis depend on the vagus nerve-to spleen axis we performed a series of experiments with mice subjected to vagotomy (VXP), splenic neurectomy (NRX) and/or splenectomy (SPX). VXP resulted in exacerbation of disease severity (Figure 2) and this effect was abolished in mice simultaneously subjected to SPX (Figure 2). The beneficial effects of McN-A-343 on the macroscopic makers and colon length were abolished in mice with VXP, SPX, or VXP and SPX (Figure 2). NRX resulted in comparable to VXP exacerbation of disease severity (Figure 2) and this effect was abolished in mice simultaneously subjected to SPX (Figure 2). Furthermore, the beneficial effects of McN-A-343 were abrogated in mice with NRX, SPX or NRX and SPX (Figure 2). Similar effects were observed in the context of DSS colitis in the absence or presence of the vagus and splenic nerves (Table 1).

**Table 1:** Central administration of a M1mAchR agonist alleviates the severity of Dextran sulfate sodium (DSS 5%)–induced colitis.

	DSS 5%					
Markers	Saline	McN-A-343	VXP	VXP + McN-A-343	NRX	NRX + McN-A-343
Macroscopic score (AU)	4.8±0.3	3.4±0.4^a^	7.9±0.6^a^	8.3±0.33^a^^,^ b	8.4±0.43^a^	7.8±0.7^a^^,^ c
Colon length (cm)	6.3±0.4	7.6±0.6^a^	5.1±0.3^a^	5.4±0.3^a^^,^ b	4.9±0.4^a^	5.1±0.6^a^^,^ c
SAP pg/ml (serum)	54.1±4.4	37.8±2.2^a^	84.2±2.7^a^	87±2.4^a^^,^ b	86.9±3.1^a^	81.1±5.1^a^^,^ c
IFN-γ pg/mg protein (colon)	40.9±2.9	18.7±1.3^a^	85.7±3.6^a^	78.3±3.5^a^^,^ b	88±4^a^	90.5±6.7^a^^,^ c
IL-17 pg/mg protein (colon)	64±6.6	13.7±2.5^a^	141.4±8^a^	145.4±8.2^a^^,^ b	163.8±15^a^	164.7±17^a^^,^ c
IL-12p70 pg/mg protein (colon)	138.6±3.7	81.4±5.1^a^	219.2±5.4^a^	230.2±7.8^a^^,^ b	231.6±8.8^a^	241.4±8.7^a^^,^ c
IL-23 pg/mg protein (colon)	67.3±4.4	34.8±6.7^a^	153.4±6.5^a^	161.3±5.5^a^^,^ b	156±7.6^a^	165.1±3.8^a^^,^ c
IL-4 pg/mg protein (colon)	29.4±2.3	25.1±4.4	30.1±3.3	31.6±2.48	25±4.8	27.2±3.3
IL-12p70 pg/ml (DC medium)	109.3±12.8	43.4±10.2^a^	198.5±7.5^a^	194±6.4^a^^,^ b	171±18^a^	183.7±9.1^a^^,^ c
IL-23 pg/ml (DC medium)	83.8±3.1	12.87±1.8^a^	142.6±14.3^a^	165.2±12.6^a^^,^ b	143.1±6.4^a^	150.6±8.2^a^^,^ c
IFN-γ pg/ml (DC:T cell co-culture medium)	1586±165	846±44^a^	2591±159^a^	2714±165^a^^,^ b	2637±125^a^	2728±134^a^^,^ c
IL-17 pg/ml (DC:T cell co-culture medium)	287.7±15.4	142.2±6.9^a^	864.6±48^a^	794.9±56.1^a^^,^ b	821.7±40.6^a^	803.7±55^a^^,^ c
IL-4 pg/ml (DC:T cell co-culture medium)	36.8±6	44.2±6.5	45±3.8	44.1±7.6	48.4±7.9	41.5±8.9

Vagotomy (VXP) or splenic neurectomy (NRX) were performed 10 days prior to initiating McN-A-343 (5 ng/kg/day, i.c.v.) treatment and/or colitis induction as described in Material and Methods. Macroscopic score, colon length, serum amyloid protein (SAP), colonic interferon-gamma (IFN-γ), interleukin (IL)-17, IL-12p70, IL-23 and IL-4. IL-12p70 and IL-23 release from splenic CD11c^+^ dendritic cells (DC). Splenic CD11c^+^ DCs were isolated from McN-A-343 (5 ng/kg/day, i.c.v. for 6 days)-treated groups of colitic mice subjected to sham-operation, vagotomy (VXP) or splenic neurectomy (NRX) on day 5 post-colitis induction. Effect of McN-A343 (*in vivo*) treatment on splenic CD11c^+^ DCs function and sequential CD4^+^ CD25^−^T cell activation. Splenic CD11c^+^ DCs isolated from different colitic group were cultured in for 24 h before being co-cultured with CD4^+^CD25^−^T cells isolated from naïve mice. The levels of IFN-γ and IL-17 and IL-4 were measured in media at 24 hrs.

a *P*<0.05 as compared to sham-saline-DSS-treated group,

b *P*<0.05 as compared to VXP-DSS-treated group or NRX-DSS-treated group.

c *P*<0.05 as compared to NRX-DSS-treated group.

### Central muscarinic cholinergic activation decreases serum SAP level and colonic Th1/Th17 cytokine release via the vagus nerve and the splenic nerve

**Figure 3:**
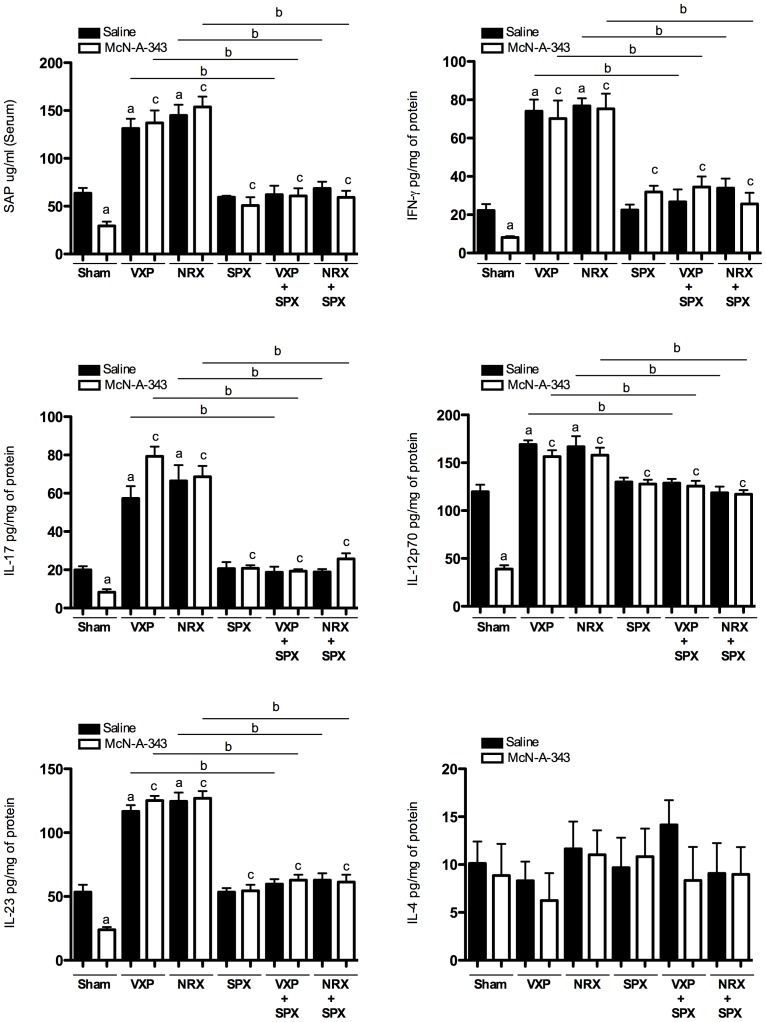
Central administration of a M1mAchR agonist alleviates the severity of 2, 4-dinitrobenzene sulfonic acid (DNBS)–induced colitis through vagus nerve and splenic nerve signaling to the spleen. Vagotomy (VXP) and/or splenectomy (SPX), splenic neurectomy (NRX) and/or splenectomy (SPX) were performed 10 days prior to initiating McN-A-343 (5 ng/kg/day, i.c.v.) treatment and/or colitis induction as described in Material and Methods. *Sham represents data obtained in sham SPX mice, because no significant differences were determined between this group and any other sham group of animals; ***A***: Serum amyloid protein (SAP); ***B***: Colonic interferon-gamma (IFN-γ); ***C***: Colonic interleukin (IL)-17; ***D***: Colonic IL-12p70; ***E***: Colonic IL-23 and ***F***: Colonic IL-4. Values are shown as means ± SEM. Samples were collected on day 3 post-DNBS induction; mice per group ≥8. ^a^ *P*<0.05 as compared to sham-saline-DNBS-treated group, ^b^ *P*<0.05 as compared to VXP-DNBS-treated group or NRX-DNBS-treated group respectively, ^c^ *P*<0.05 as compared to sham-McN-A-343-DNBS-treated group.

To determine the regulatory effect on colonic Th1-Th2 and Th17 cytokine profile, IFN-γ, IL-17, IL-12p70 IL-23 and IL-4 were studied. McN-A-343 (5 ng/kg/day) treatment, started one day before induction of DNBS-colitis decreased all colonic inflammatory markers studied. Decreased severity of colitis in McN-A-343-treated mice as compared to saline-treated mice was evident by the 2.1-fold decrease in the SAP levels (Figure 3A). The increase in colonic in IFN-γ was 2.7-fold lower (Figure 3B) and the up regulation of IL-17 (Figure 3C), IL-12p70 (Figure 3D), and IL-23 (Figure 3E) were respectively 2.4-fold, 3.1-fold and 2.2-fold lower. Same pattern of results were found when IL-1 β, IL-6 and TNF-α were studied (Figure S1). No significant changes were detected in IL-4 levels (Figure 3F). Similar cytokine alterations were determined in the context of DSS colitis (Table 1).

VXP resulted in exacerbation of disease severity (Figure 3) and this effect was abolished in mice simultaneously subjected to SPX (Figure 3). The beneficial effects of McN-A-343 on SAP cytokine levels and cytokine were abolished in mice with VXP, SPX, or VXP and SPX (Figure 3, Figure S1). NRX resulted in comparable to VXP exacerbation of disease severity (Figure 3, Figure S1) and this effect was abolished in mice simultaneously subjected to SPX (Figure 3, Figure S1). Furthermore, the beneficial effects of McN-A-343 were abrogated in mice with NRX, SPX or NRX and SPX (Figure 3, Figure S1). Similar effects were observed in the context of DSS colitis in the absence or presence of the vagus and splenic nerves (Table 1).

### Central cholinergic activation decreases splenic CD11c^+^ DCs IL-12p70 and IL-23 release via the vagus nerve and the splenic nerve

**Figure 4:**
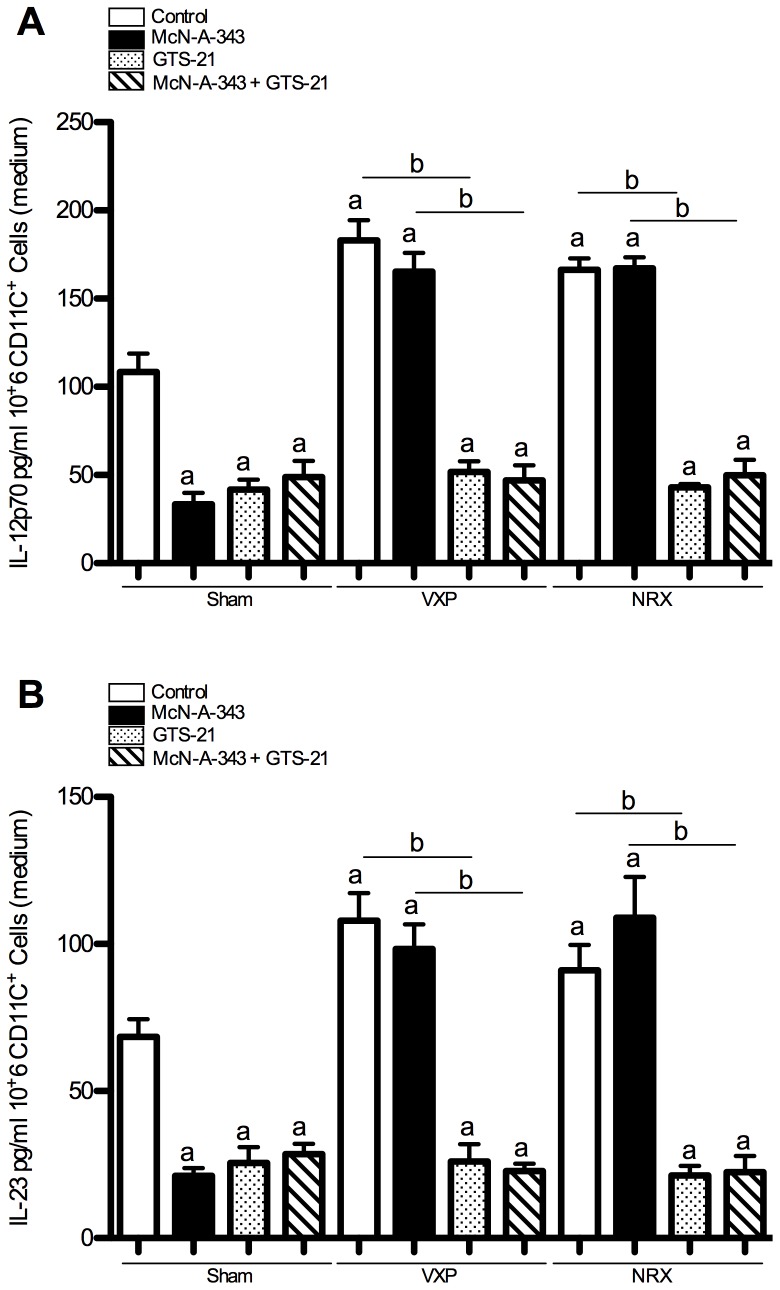
Central administration of a M1mAChR agonist alleviates splenic CD11c^+^ dendritic cells (DCs) interleukin (IL)-12p70 and IL-23 release in the context of 2, 4-dinitrobenzene sulfonic acid (DNBS)–induced colitis through vagus nerve and splenic nerve signaling to the spleen. ***A***: IL-12p70 and ***B***: IL-23 production from splenic CD11c^+^ DCs. Splenic CD11c^+^ DCs were isolated from control and McN-A-343 (5 ng/kg/day, i.c.v. for 4 days)-treated groups of colitic mice subjected to sham-operation, vagotomy (VXP) or splenic neurectomy (NRX) on day 3 post-DNBS induction incubated *ex vivo* or not with GTS-21 (a specific α7nAChR agonist, 100 µM). IL-12p70 and IL-23 were measured in media at 24 hrs following treatments. Values are shown as means ± SEM, 3 independent experiments with 4 mice per group. ^a^ *P*<0.05 as compared to DNBS-control group, ^b^ *P*<0.05.

To provide further insight into the cellular mechanisms mediating cholinergic anti-inflammatory effects in the DNBS model of colitis, we studied the role of splenic CD11c^+^ DCs. We found a significant decrease of IL-12p70 and IL-23 in supernatants of splenic CD11c^+^ DCs isolated from colitic McN-A-343-infused mice (Figure 4) as compared to colitic vehicle-treated controls. Conversely, VXP or NRX treatments increased the two cytokines and no beneficial effect of McN-A-343 infusion was found in the absence of an intact vagus nerve or splenic nerve (Figure 4). Similar effects were observed in the context of DSS colitis in the absence or presence of the vagus and splenic nerves (Table 1). No effect was detected when IL-4 was studied (data not shown).

DC express α7nAChR, to confirm the role of the α7nAChR at the splenic level, we isolated splenic CD11c^+^ DCs from sham-operated, VXP or NRX DNBS-colitic mice. In colitic mice with VXP or NRX, addition of GTS-21 (a specific α7nAChR agonists, 100 µM) in the culture medium significantly decreased the production of IL-12p70 and IL-23 (Figure 4). Conversely, addition of methyllycaconitine (a specific α7nAChR antagonist, 10 µM) in the medium reversed the beneficial effect of the GTS-21 treatment (IL-12p70: Sham 99±10; VXP 178±14; NRX 165±17 pg/ml and IL-23: Sham 52±9; VXP 103±12; NRX 96±16 pg/ml). No effect of methyllycaconitine treatment was detectable in Sham, VXP and NRX groups not treated with GTS-21. No synergistic affect was observed in CD11c^+^ DCs isolated from colitic McN-A-343-infused mice and treated *in vitro* with GTS-21. No effect on splenic CD11c^+^ DCs proliferation was observed (data not shown).

### Central cholinergic activation decreases splenic CD11c^+^ DCs IL-12p70 and IL-23 release via the NF-kB pathway

**Figure 5:**
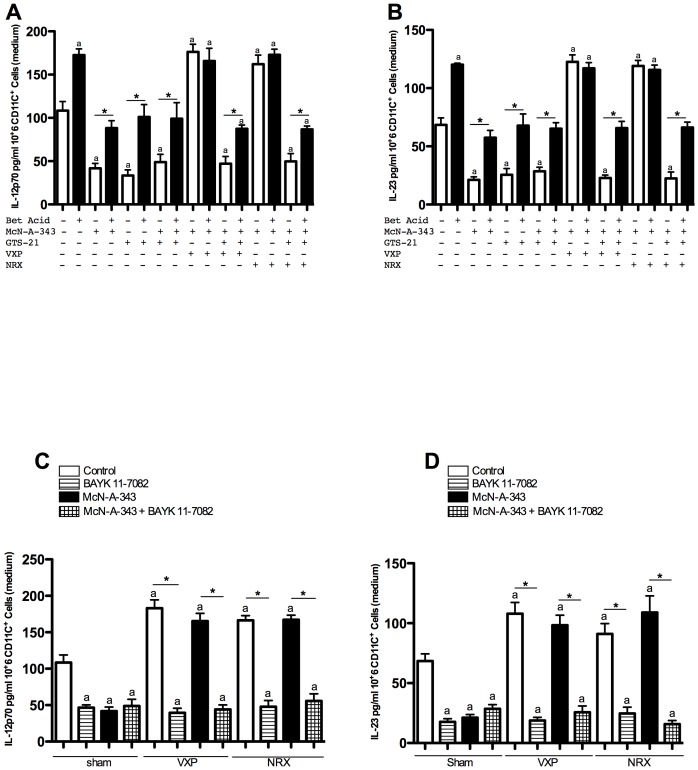
Implication of the NF-kB pathway in splenic CD11c^+^ dendritic cells (DCs) cytokine release in the context of 2, 4-dinitrobenzene sulfonic acid (DNBS)–induced colitis. Interleukin (IL)-12p70 and IL-23 production from splenic CD11c^+^ DCs. Splenic CD11c^+^ DCs were isolated from control and McN-A-343 (5 ng/kg/day, i.c.v. for 4 days)-treated groups of colitic mice subjected to sham-operation, vagotomy (VXP) or splenic neurectomy (NRX) on day 3 post-DNBS induction incubated *ex vivo* or not with GTS-21 (a specific α7nAChR agonist, 100 µM) or with ***A, B***: betulinic acid (a specific NF-κB activator, 10 µM) or ***C, D***: BAY 11-7082 (a specific NF-κB inhibitor, 10 µM). IL-12p70 and IL-23 were measured in media at 24 hrs following treatments. Values are shown as means ± SEM, 3 independent experiments with 4 mice per group. ^a^ *P*<0.05 as compared to DNBS-control group, ^*^ *P*<0.05.

Previously, an increase of splenic ACh levels after central activation of the CAP accompanied by a decrease of splenic CD11c^+^ DCs IL-12p40 release has been demonstrated [9]. To provide insight into the transduction pathway by which central cholinergic activation decreases IL-12p70 and IL-23 in splenic CD11c^+^ DCs, we pre-treated isolated splenic CD11c^+^ DCs from different groups with an NF-κB activator (betulinic acid) or an inhibitor (BAY 11-7082) for 24 hours. Using the DNBS model, the beneficial effect of the *in vivo* McN-A-343 infusion or *ex vivo* treatment with GTS-21 was partially reversed in the presence of betulinic acid (Figure 5A, B). Conversely, the deleterious effect of VXP ad NRX was abolished in the presence of BAY 11-7082 (Figure 5C, D). These results highlight the importance of the NF-κB pathway in splenic CD11c^+^ DCs as a mediating event of IL-12p70 and IL-23 release during colitis and its suppression by central muscarinic cholinergic activation of the vagus nerve -to spleen axes.

### Central cholinergic activation regulates splenic CD11c^+^ DCs priming of CD4^+^CD25^−^ T cells via the vagus nerve and splenic nerve

**Figure 6:**
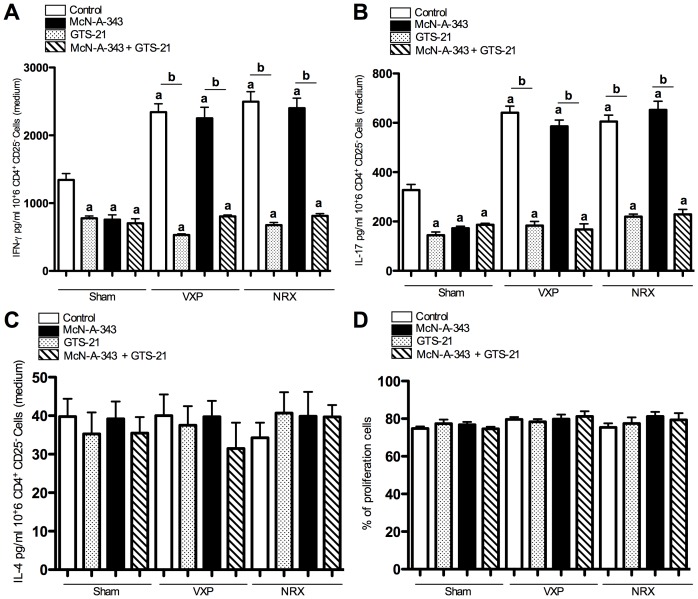
Role of the CD11c^+^ dendritic cells (DCs) in CD4^+^CD25^−^ T cells priming in the context of 2, 4-dinitrobenzene sulfonic acid (DNBS)–induced colitis. Effect of McN-A343 (*in vivo*) and GTS-21 (*ex vivo*) treatments on splenic CD11c^+^ DCs function and sequential CD4^+^CD25^−^T cell activation. Splenic CD11c^+^ DCs were isolated from control and McN-A-343 (5 ng/kg/day, i.c.v. for 4 days)-treated groups of colitic mice subjected to sham-operation, vagotomy (VXP) or splenic neurectomy (NRX) on day 3 post-DNBS induction and were cultured in the presence or absence of GTS-21 (a specific α7nAChR agonist, 100 µM) for 24 hrs before medium was washed and co-cultured with CD4^+^/CD25^−^ T cells isolated from naïve mice. The level of ***A***: Interferon-gamma (IFN-γ); ***B***: Interleukin (IL)-17 and ***C***: IL-4 were measured in media at 24 hrs; ***D***: CD4^+^CD25^−^ T cells proliferation. ^a^ *P*<0.05 as compared to DNBS-control group, ^b^ *P*<0.05, n = 8, data are representative of 3 independent experiments with quadruplicated cultures, mean ± SEM.

Recent data demonstrated that the central cholinergic activation decreases splenic CD11c^+^ DCs cytokine production and MHC class II marker and that this effect was dependent on neural signals along the vagus and the splenic nerve [9]. To demonstrate the importance of the DC cytokine release *vs* delivery of co-stimulatory signals, we studied the T-cell priming function of splenic CD11c^+^ DCs. To determine the contribution of CD4^+^CD25^−^ T cells to the decreased cytokines production observed in the spleen following activation of the CAP during DNBS-induced colitis, we isolated CD4^+^CD25^−^ T cells from the spleen of naïve animals and determine the splenic CD11c^+^ DCs priming of CD4^+^CD25^−^ T cells and their subsequent cytokine release in the presence of anti-CD3/Cd28 Abs. CD4^+^CD25^−^ T cells, isolated from naïve mice were co-cultured for 24 h with splenic CD11c^+^ DCs isolated from DNBS-colitic mice *in vivo* treated with McN-A-343. CD4^+^CD25^−^ T cells released significantly lower amount of IFN-γ (Figure 6A), IL-17 (Figure 6B) as compared to CD4^+^CD25^−^ T cells co-cultured with splenic CD11c^+^ DCs isolated from *in vivo* saline-treated colitic mice. Conversely, naïve CD4^+^CD25^−^ T cells released significantly more IFN-γ and IL-17 when co-cultured with splenic CD11c^+^ DCs isolated from vagotomized or neurectomized colitic mice. Similar effects were observed in the context of DSS colitis in the absence or presence of the vagus and splenic nerves (Table 1). No beneficial effect of McN-A-343 infusion was found in the absence of an intact vagus and splenic nerve. None of the treatments had an effect on the IL-4 levels (Figure 6C).

To evaluate the role of the α7nAChR we used splenic CD11c^+^ DCs isolated from sham-operated, VXP, and NRX-colitic mice. At all conditions, DC pretreatment with GTS-21 significantly decreased the production of IFN-γ and IL-17. No synergistic affect was observed in DCs isolated from colitic McN-A-343 treated mice and treated *in vivo* with GTS-21. No drug effect on T-cell proliferation was observed within a 24 h period of time (Figure 6D).

### Central cholinergic activation regulates splenic CD11c^+^ DCs priming of CD4^+^CD25^−^ T cells via IL-12 and IL-23

**Figure 7:**
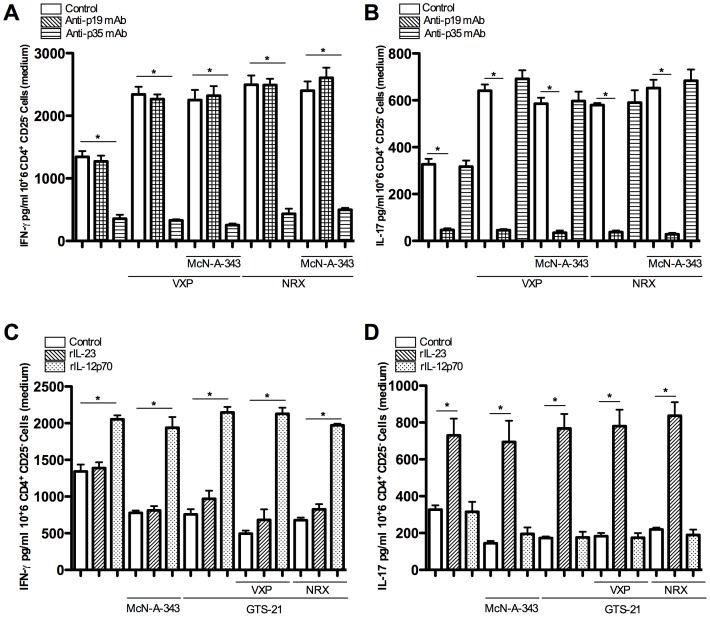
Implication of the interleukin (IL)-12p70 and IL-23 pathways during CD4^+^CD25^−^ T cells priming by splenic CD11c^+^ dendritic cells (DCs) in the context of 2, 4-dinitrobenzene sulfonic acid (DNBS)–induced colitis. Splenic CD11c^+^ DCs isolated from different colitic group were cultured in for 24 h before being co-cultured with CD4^+^/CD25^−^ T cells isolated from naïve mice in the presence or absence of ***A, B***: anti-p19 mAb (10 ug/ml^−1^) or anti-p35 mAb (10 ug/ml^−1^); ***C, D***: recombinant (r)IL-12p70 (25 ng/ml^−1^) or rIL-23 (25 ng/ml^−1^) protein. Supernatant were collected after 24 h. The levels of interferon-gamma (IFN-γ) and IL-17 in the culture supernatant were investigated at 24 hrs. *P<0.05, data are representative of 3 independent experiments with quadruplicated cultures, mean ± SEM.

The p40 subunit is shared by the IL-12 and IL-23 cytokine [26], [27]. To determine the mechanisms by which central cholinergic activation decrease T-cell priming, co-culture we treated with anti p19-mAb or p35-mAb. In the presence of anti p19-mAb, the deleterious effect of VXP ad NRX was abolished in terms of the IL-17 cytokine profile (Figure 7B) whereas in the presence of anti p35-mAb only IFN-γ levels were affected (Figure 7A). Conversely, addition of IL-12p70 or IL-23 recombinant proteins restore the level of IFN-γ and IL-17 in CD4^+^CD25^−^ T cells conditioned with splenic CD11c^+^DCs isolated from colitic mice treated with McN-A-343 or GTS-21 (Figure 7C, D).

## Discussion

Mucosal inflammation in IBD is accompanied by autonomic nervous system dysfunction with decreased vagus nerve activity [28]. It is recognized that this autonomic dysfunction plays an important role in motility and immune response dysregulation during IBD [29], [30]. Over the last decade it has been demonstrated that the vagus nerve-based CAP has a major anti-inflammatory function in different inflammatory conditions including colitis and in models of UC and CD [9], [12], [31]. While the spleen plays an important mediating role in the cholinergic anti-inflammatory pathway [10], , at certain experimental conditions of intestinal inflammation this physiological mechanism has been shown to require non neural signaling to spleen [34]. Thus, the precise mechanisms mediating the impact of the CAP on specific intestinal inflammation and their cellular and intracellular aspects remain to de elucidated.

Here, we show that brain mAChR mediated cholinergic activation results in a decreased susceptibility to experimental colitis; this effect is vagus nerve- and splenic nerve-dependent and mediated at a cellular level by a specific interaction between CD11c^+^ DCs and CD4^+^/CD25^−^ T cells.

The DNBS-induced colitis is a widely used model of CD [21] and is a T-cell dependent model. Depending on the concentration, the duration, and frequency of DNBS administration, animals may develop acute or chronic colitis or even colitis-induced dysplastic lesions. Dysregulated innate and adaptive immune responses are shown to be important components in colitis pathogenesis in this model. Recently we demonstrated that brain stimulation of the CAP alleviates the disease severity and suppresses colonic proinflammatory cytokine (IL-6, IL-1β and TNF-α) levels in experimental DSS-induced colitis. Here, after demonstrating the beneficial effect of central cholinergic activation by a M1mAChR agonist in DNBS-colitis, we demonstrate that this effect is accompanied by a downregulation of IFN-γ and IL-17, IL-1 β, IL-6 and TNF-α levels in colonic tissues. The beneficial effect of the treatment was completely abolished in the absence of the vagus or the splenic nerves. As the vagus nerve stimulates gastrointestinal motility, we verified that pharmacological VN stimulation did not increase diarrhea in our study. In control group and at the dose used, stool consistency was not affected.

The lack of significant effects of the McN-A-343 infusion on colonic level of IL-4 in mice DNBS-induced colitis observed in our study suggests that IL-4 do not play a role in mediating the beneficial effects of cholinergic modalities on colitis severity, as in the context of colitis IL-4 is known to decrease T cell priming [35]. This observation is in line with previous data demonstrating the minor role of vagotomy on IL-4 in colitis [36]. Alternatively, using a lymphocyte-independent self-limiting model of erosive colonic injury and inflammation through oral administration of DSS^22, 23^, we demonstrated similar results.

Although previous studies have implicated a key role of splenic DCs [9], [37] and T cells [32] in the context of the CAP, it was not clear whether alterations in antigen presenting cell response and T cells priming play any mediating role in the cholinergic regulation of gut inflammation. We have recently demonstrated an increase of splenic IL-12p40 after pharmacological stimulation of the vagus nerve [9]. The fact that the p40 subunit is commonly shared by IL-12p70 and IL-23 [26], [27] prompted us to study IL-12p70 and IL-23 levels. Alleviated inflammation in DNBS-induced colitis was accompanied by reduced level of IL-12p70 and IL-23 in the colon and from splenic CD11c^+^ DCs isolated from colitic mice treated with McN-A-343. Conversely, we also observed that colon and CD11c^+^ DCs isolated from group without an intact vagus nerve and splenic nerve produced significantly more IL-12p70 and IL-23 and that the beneficial effect of the MCN-A-343 treatment was abolished in the absence of the vagus and splenic nerves. Moreover, *in vitro* stimulation with a specific α7nAChR agonist decreased IL-12p70 and IL-23 production of CD11c^+^ DCs isolated from both groups. This is in accordance with data demonstrating a downregulation of IL-12p70 and IL-23 in polymorphonuclear neutrophils treated with nicotine [38], the close relation between the splenic nerve and the DC population within the spleen [39] and splenic Ach release after splenic nerve stimulation [10]. In our models, it is possible that mesenteric lymphoid or lamina propria DCs may also be involved in mediating cholinergic effects in the context of colitis, but our data demonstrate the role of splenic CD11_C_^+^ cells. However, the possible contribution of other circulating immune cell expressing the α7nAChR need also be considered, as it has been reported that α7AChR are expressed not only on DCs but also on other immune cells including monocytes [40], [41], polymorphonuclear neutrophils [42], B cells [43] and T cells [44]. Further experiments need to be conduced to decipher the exact role of these cell-types.

NF-κB, an important transcription factor mediating the proinflammatory cytokine production is attributed to the initiation and progression of colonic inflammation in mice and humans [45], [46]. nAChRs are expressed on antigen presenting cell including macrophages and DCs [40], and ACh and nicotine inhibit TNF-α and NF-κB production from lipopolysaccharide (LPS)-stimulated human macrophages and splenocytes [47]. In the context of polymorphonuclear neutrophil activation, it has also been described that GTS-21 can inhibit phosphorylation and subsequent degradation of I-κB, which is likely to suppress NF-κB activation [48]. As nAChRs are expressed on DCs [40], we tested the role of the intracellular NF-κB pathway. Increased production of IL-12p70 and IL-23 by DCs in VXP- and NRX mice was significantly inhibited in the presence of the NF-κB pathway inhibitor. Moreover, in the presence of NF-κB pathway activator the levels of both cytokines were increased in CD11c^+^ DCs isolated from stimulated groups. *In vitro* stimulation with GTS-21 decreased IL-12p70 and IL-23 production of CD11c^+^ DCs isolated from both group and this effect was abolished in the presence of the NF-κB activator. Together, these findings indicate that deactivation of the NF-κB signaling pathway is essential for pharmacological cholinergic stimulation to inhibit DCs activation, which is critical in the pathogenesis of colitis in this model. This corroborates data demonstrating that *in vitro* mature spleen DCs that are exposed to nicotine produce decreased levels of IL-12 and display reduced ability to induce T cell responses [15].

Our results further demonstrate that CD11c^+^ DCs isolated from DNBS-colitic mice treated with McN-A-343 have significantly reduced ability to stimulate naïve CD4^+^CD25^−^ T cells *in vitro* to produce IFN-γ and IL-17 as compared to the CD11c^+^ DCs isolated from colitic non-treated mice. Moreover, our data demonstrate, that using the antiCD3/CD28 stimulation model, the cytokine released by the DC are sufficient to direct T-cell priming. This observation provides direct evidence of the importance of DCs in colitis. It is in agreement with studies in the context of experimental colitis [16] demonstrating amelioration of colitis by depletion of DCs after administration of diphtheria toxin in CD11c-DT receptor transgenic mice [17]. The clinical relevance of these findings is substantiated by previous data demonstrating that IBD is characterized by increased expression of innate (IL-12, IL-23) and adaptive (IFN-γ, IL-17) proinflammatory cytokines [5], and Th1, Th17 polarization is mainly controlled by the IL-12p70 and IL-23 cytokine family produced by activated DCs [49]. Increased numbers of MDC8^+^ monocytes, which are the precursors of mucosal DC populations, are found in patients with IBD and, hence, anti-TNF treatment results in reduced DCs activation [50]. Moreover, in the context of IBD, DCs are increased within the lamina propria and peripheral blood [51] and DCs isolated from peripheral blood monocytes are potent immune response stimulators [52].

We also further identify the mediating role of IL-12p70 and IL-23 on DCs/T cell priming. In the absence of an intact vagus or splenic nerve the presence of a specific anti-p35 Ab decreased exclusively IFN-γ release from T cells. Conversely, in the presence of anti-p19 Ab only the IL-23 levels were affected. This corroborates clinical and experimental data demonstrating an increase in IL-12p40, IL-12p70 and IL-23p19 in intestinal tissue [53], [54]. A beneficial effect of anti p40 or p19 mAbs on human and experimental colitis has been proposed [55], [56]. IL-12 and IL-23 that share the same p40 subunit may play distinct roles in colitis, depending on the mechanism involved. IL-23p19-deficient mice develop severe colitis in IL-10 deficient mice [57] and IL-12p35 deficient mice develop a mild colitis [58]. These somewhat conflicting observations suggest diverse roles of IL-12 and IL-23 in colitis depending on the model used. Here, we highlight the role of brain pharmacological activation of the CAP in controlling T cell priming *via* DC deactivation and the specific mediating role of IL-12 and IL-23. In addition, these studies show the efficacy of this approach in suppressing proinflammatory cytokine production from DCs through an NF-κB intracellular mechanism and sequential T-cell instigation.

It is possible that in addition to the spleen the vagus nerve-based CAP signals in other organs implicated in the development of colonic inflammation [59]. As in human the VN does not innervate the distal colon, is also conceivable that other, vagus nerve-related factors may play a role in the context of IBD, as the vagus nerve influences gastrointestinal motility and controls the ileocecal valve in several species [60], [61]. A central potential activation of the HPA axis associated with a decreased colonic inflammation cannot been excluded [62].

Our findings provide new insight into the mechanisms of regulation of mucosal inflammation by brain activation of the CAP. It is interesting to note that another pharmacological brain activator of the CAP - CNI-1493 (semapimod), an inhibitor of p38 mitogen-activated protein kinase [63], has now been clinically tested in patients with CD [64]. This demonstrates, that the activation of the VN in the context of the anti-inflammatory pathway might be efficient in IBD patients. A recent case report published the group of Prof. Bonaz, demonstrated the therapeutic long-term effects of low frequency vagus nerve stimulation on EEG and heart rate variability in CD. In this context the patient presented a significant clinical improvement, with a progressive decrease of the CDAI score and an endoscopic remission at month 12, associated to an increase of the parasympathetic tone [65]. Moreover, complementary medicine such as cognitive behavioral therapies or hypnosis, known to modify vagal would be of interest [66]–[69].

Our study contributes to better understanding of the pathogenesis of colitis and provides novel information related to vagus nerve-to spleen immune regulation by highlighting a key role for DCs-T-cell priming. Our findings foster consideration of the relationship between brain cholinergic activation and disease activity in patients with IBD and suggest further development of centrally-acting selective M1mAChR agonists as novel experimental therapeutics in colitis, or alternatively methods to increase the VN outflow (*e.g.* electrical VN stimulation, or alternative medicine).

## Supporting Information

Figure S1 **Central administration of a M1mAchR agonist alleviates the severity of 2, 4 dinitrobenzene sulfonic acid (DNBS)–induced colitis through vagus nerve and splenic nerve signaling to the spleen.** Vagotomy (VXP) and/or splenectomy (SPX), splenic neurectomy (NRX) and/or splenectomy (SPX) were performed 10 days prior to initiating McN-A-343 (5 ng/kg/day, i.c.v.) treatment and/or colitis induction as described in Material and Methods. *Sham represents data obtained in sham SPX mice, because no significant differences were determined between this group and any other sham group of animals; ***A***: Colonic interleukin (IL)-17; ***B***: Colonic IL-16; ***C***: Colonic Tumor necrosis factor (TNF)-alpha. Values are shown as means ± SEM. Samples were collected on day 3 post-DNBS induction; mice per group 8. ^a^ *P*<0.05 as compared to sham-saline-DNBS-treated group, ^b^ *P*<0.05 as compared to VXP-DNBS-treated group or NRX-DNBS-treated group respectively, ^c^ *P*<0.05 as compared to sham-McN-A-343-DNBS-treated group.
(TIFF)
