# Supplementary figures and images for: Central Muscarinic Cholinergic Activation Alters Interaction between Splenic Dendritic Cell and CD4+CD25- T Cells in Experimental Colitis
Source: PLoS One. 2014 Oct 8;9(10):e109272. doi: 10.1371/journal.pone.0109272 (PMC4190311; doi:10.1371/journal.pone.0109272)

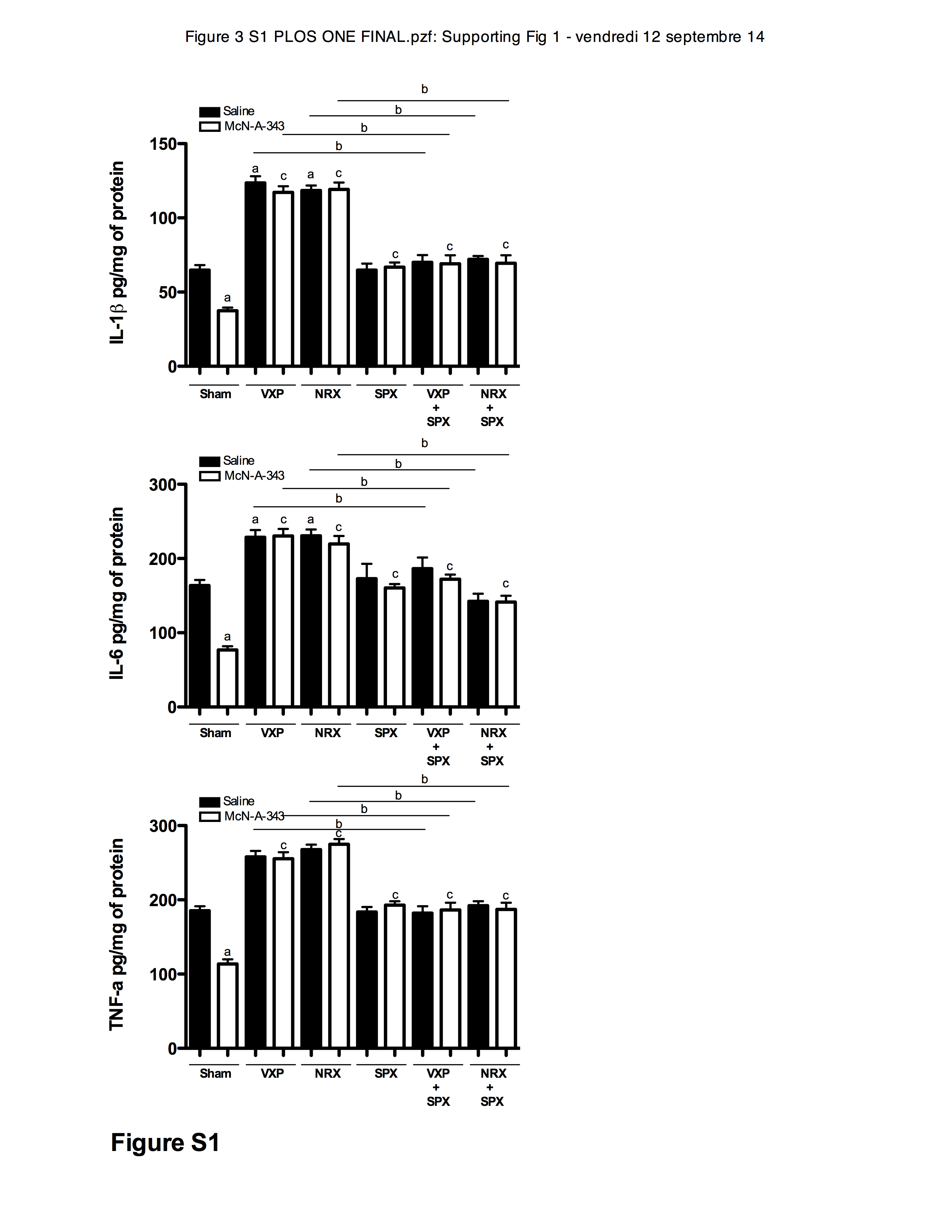

Supplement: Figure S1 — Central administration of a M1mAchR agonist alleviates the severity of 2, 4 dinitrobenzene sulfonic acid (DNBS)–induced colitis through vagus nerve and splenic nerve signaling to the spleen. Vagotomy (VXP) and/or splenectomy (SPX), splenic neurectomy (NRX) and/or splenectomy (SPX) were performed 10 days prior to initiating McN-A-343 (5 ng/kg/day, i.c.v.) treatment and/or colitis induction as described in Material and Methods. *Sham represents data obtained in sham SPX mice, because no significant differences were determined between this group and any other sham group of animals; A: Colonic interleukin (IL)-17; B: Colonic IL-16; C: Colonic Tumor necrosis factor (TNF)-alpha. Values are shown as means ± SEM. Samples were collected on day 3 post-DNBS induction; mice per group 8. a P<0.05 as compared to sham-saline-DNBS-treated group, b P<0.05 as compared to VXP-DNBS-treated group or NRX-DNBS-treated group respectively, c P<0.05 as compared to sham-McN-A-343-DNBS-treated group. (TIFF) [file pone.0109272.s001.tiff]
